# Jejunal Metastasis from Malignant Pleural Mesothelioma during Long-Term Nivolumab Therapy: A Case Report and Literature Review

**DOI:** 10.70352/scrj.cr.25-0774

**Published:** 2026-02-13

**Authors:** Hitoshi Minagi, Hideki Aoki, Mikoto Nosaka, Toshihiro Ogawa, Megumi Watanabe, Takashi Arata, Koh Katsuda, Kohji Tanakaya

**Affiliations:** 1 Department of Gastroenterological Surgery, Okayama University Graduate School of Medicine, Dentistry and Pharmaceutical Sciences, Okayama, Okayama, Japan; 2 Department of Surgery, National Hospital Organization, Iwakuni Clinical Center, Iwakuni, Yamaguchi, Japan

**Keywords:** malignant pleural mesothelioma, jejunal metastasis, small-bowel metastasis, immune-checkpoint inhibitors

## Abstract

**INTRODUCTION:**

Gastrointestinal metastasis from malignant pleural mesothelioma (MPM) is exceedingly rare and lacks a standardized diagnostic work-up.

**CASE PRESENTATION:**

An 87-year-old man with asbestos exposure and a 5-year history of left-sided MPM, radiographically stable on long-term nivolumab therapy, presented with melena and anemia. Contrast-enhanced CT revealed a circumferential jejunal lesion near the duodenojejunal flexure with enlarged mesenteric nodes. Double-balloon enteroscopy confirmed a 40-mm obstructive tumor, but biopsies were non-diagnostic. Partial jejunectomy with targeted mesenteric lymph-node dissection was performed. Histology with a mesothelioma-oriented immunohistochemical panel—negative for CEA and BerEP4 and positive for broad-spectrum cytokeratins—supported a diagnosis of metastatic MPM; nodal metastases were present. Given his age, no further systemic therapy was administered; the patient died 20 months postoperatively from progression of MPM.

**CONCLUSIONS:**

In MPM receiving immune-checkpoint inhibitor therapy, unexplained gastrointestinal bleeding should prompt comprehensive gastrointestinal evaluation, including small-bowel assessment. Surgery can secure symptom relief and a definitive diagnosis.

## Abbreviations

18F-FDG PET
: ^18^F-fluorodeoxyglucose PET
DBE
: double-balloon enteroscopy
ICI
: immune-checkpoint inhibitor
IHC
: immunohistochemistry
MPM
: malignant pleural mesothelioma

## INTRODUCTION

MPM is an aggressive malignancy with a poor prognosis, typically spreading by contiguous invasion; clinically apparent distant metastases are uncommon. Gastrointestinal involvement is rare, and small-bowel metastasis is particularly uncommon. We report a resected case of jejunal metastasis from MPM and highlight diagnostic and surgical considerations.

## CASE PRESENTATION

**Fig. 1:**
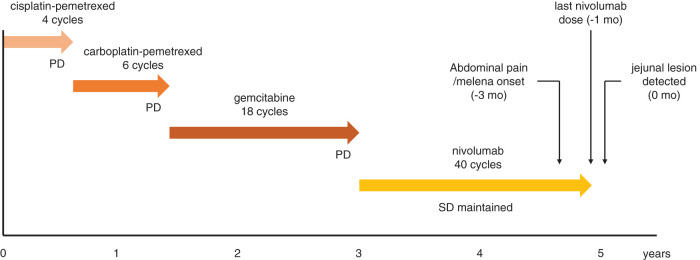
Clinical course. Timeline of systemic therapies and disease status. PD followed cisplatin–pemetrexed, carboplatin–pemetrexed, and gemcitabine, whereas SD was maintained during nivolumab therapy. Symptom onset, last nivolumab dose, and detection of the jejunal lesion are indicated. PD, progressive disease; SD, stable disease

An 87-year-old former seaman with occupational asbestos exposure had been diagnosed with left-sided MPM 5 years earlier. The primary MPM was diagnosed as epithelioid mesothelioma, showing positivity for cytokeratin AE1/AE3 and mesothelial markers (calretinin and D2-40) and negativity for TTF-1, CEA, and desmin. He had received multiple systemic regimens, including 4 cycles of cisplatin–pemetrexed, 6 cycles of carboplatin–pemetrexed, and 18 cycles of gemcitabine, followed by long-term nivolumab therapy (40 cycles over 21 months). During nivolumab therapy, MPM remained radiographically stable, and nivolumab was continued until approximately 1 month before the gastrointestinal evaluation described below (**Fig. 1**). Prior malignancies included rectal, bladder, and prostate cancers. Over the preceding 3 months, he developed abdominal discomfort and melena; progressive anemia prompted evaluation.

**Fig. 2:**
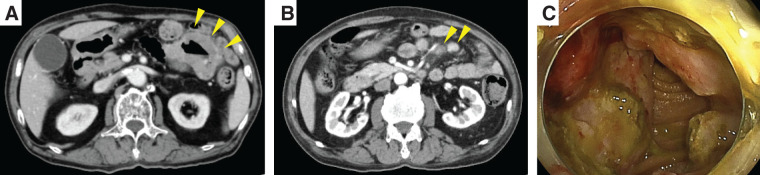
Images of contrast-enhanced CT and DBE. (**A**) CT images showing a circumferential, contrast-enhancing jejunal lesion near the duodenojejunal flexure (yellow arrowheads). (**B**) Multiple enlarged mesenteric lymph nodes along the tumor-feeding vessels (yellow arrowheads). (**C**) DBE demonstrating a 40-mm, circumferential, near-obstructive jejunal tumor just distal to the duodenojejunal flexure. Distal scope passage was impeded. DBE, double-balloon enteroscopy

Contrast-enhanced CT showed a circumferential, contrast-enhancing jejunal lesion near the duodenojejunal flexure with enlarged mesenteric lymph nodes (**Fig. 2A**, **2B**). DBE confirmed a 40-mm, circumferential, near-obstructive lesion just distal to the flexure (**Fig. 2C**). Endoscopic biopsies were non-diagnostic; immunostaining of the biopsy specimens for mesothelial markers was negative. Serum epithelial tumor markers were not elevated, whereas serum hyaluronic acid was increased (115 ng/mL; reference <50 ng/mL). Given symptom progression, ^18^F-FDG PET/CT was deferred.

**Fig. 3:**
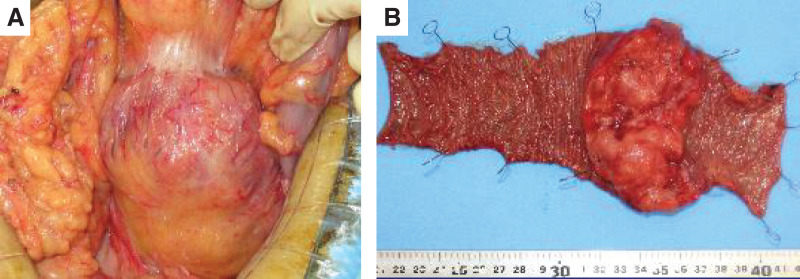
Intraoperative images and resected specimen. (**A**) Intraoperative photograph showing an inflamed jejunal mass adherent to the transverse mesocolon. (**B**) Resected specimen of the jejunum with a circumferential lesion; the mucosal surface appears relatively smooth.

Surgery was performed for diagnosis and improvement of anemia and abdominal pain. A jejunal tumor with surrounding inflammatory change was identified approximately 10 cm distal to the duodenojejunal flexure, adhered to the transverse mesocolon (**Fig. 3**). Partial jejunectomy with targeted mesenteric lymph-node dissection was performed.

**Fig. 4:**
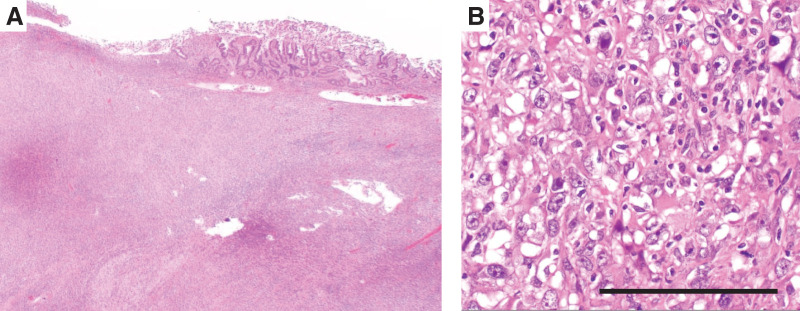
H&E staining. (**A**) Low-power view showing transmural infiltration with focal mucosal preservation. (**B**) High-power view showing ovoid to spindle tumor cells with enlarged, irregular nuclei. Scale bar: 200 μm. H&E, hematoxylin–eosin

**Fig. 5:**
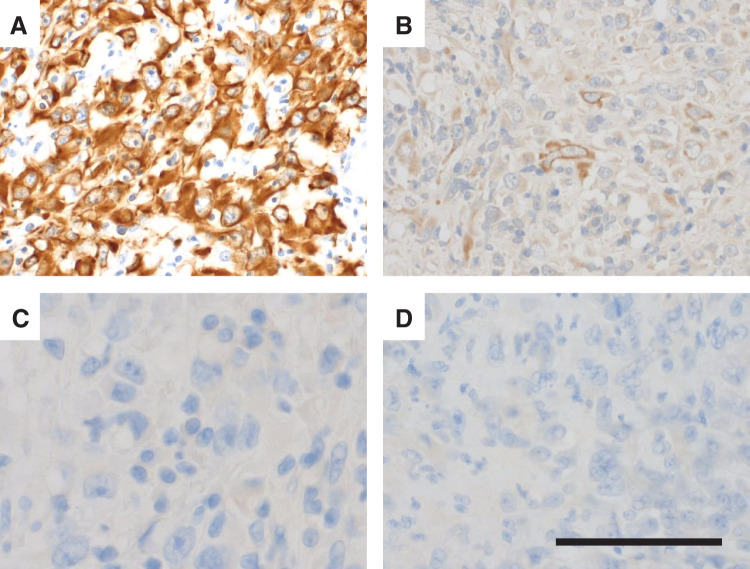
IHC. (**A**) CAM5.2 positive. (**B**) AE1/AE3 weakly positive. (**C**) CEA negative. (**D**) Ber EP4 negative. These findings indicate positivity for broad-spectrum cytokeratins with epithelial markers negative, supporting metastatic MPM in the appropriate clinical context. Scale bar: 200 μm. IHC, immunohistochemistry; MPM, malignant pleural mesothelioma

Histopathology showed transmural infiltration of atypical cells with focal mucosal preservation (**Fig. 4**). IHC demonstrated partial positivity for broad-spectrum cytokeratins (CAM5.2, AE1/AE3), negativity for epithelial markers (CEA, BerEP4) (**Fig. 5**), and negativity for D2-40; calretinin and WT-1 showed only equivocal (focal/weak) staining. In the clinical context of MPM, the overall findings favored a diagnosis of jejunal metastasis from MPM, with mesenteric lymph-node metastases.

On POD 9, the patient developed ileus, which resolved with conservative management; he was discharged on POD 45. Given his age, no further systemic therapy was administered. He died 20 months postoperatively from progression of MPM.

## DISCUSSION

Although the small bowel comprises more than 75% of the gastrointestinal tract length and approximately 90% of its mucosal surface area, primary small-bowel cancers account for only about 5% of all gastrointestinal malignancies.^1)^ In an endoscopy-based review of 530 small-bowel malignancies, Hirai et al. reported gastrointestinal stromal tumors (30.4%) and primary adenocarcinoma (22.5%) as most frequent, whereas metastatic tumors accounted for 9.1%.^2)^ Common primary sites for small-bowel metastases include lung, renal, and breast cancers.^3–5)^

MPM is strongly associated with asbestos exposure. While contiguous invasion is typical, clinically apparent distant metastasis is considered uncommon. In contrast, an autopsy series by Finn et al. documented extrathoracic metastases in 55% of 318 cases—mainly lymph nodes, contralateral lung, adrenal glands, and liver.^6)^ Gastrointestinal metastasis—particularly to the small bowel—is rare. We searched PubMed through December 31, 2024, using the query “malignant pleural mesothelioma” AND “metastasis” AND (“small intestine” OR “small bowel” OR “duodenum” OR “jejunum” OR “ileum”); this English-language search identified nine cases, including ours (**Table 1**).^7–14)^ Across these reports, the median age was 65 years, all patients were male despite the overall male-to-female ratio of ~4:1 in MPM,^15)^ and the median interval to small-bowel metastasis was 9 months from MPM diagnosis; our case occurred at 60 months, the longest reported. This prolonged interval may reflect improved survival under immune-checkpoint inhibition and/or tumor biology; however, causality cannot be established.

**Table 1:** Summary of reported cases of small-bowel metastasis of MPM

	Author	Year	Age	Sex	Race	Onset	Treatment for MPM	Duration to metastases (months)	Location of metastases	Metastases to other organs	Treatment for metastases	Prognosis
1	Kakugawa^7)^	2007	62	M	Asian	Anemia	EPP	9	Jejunum, Ileum	ND	ND	ND
2	Chen^8)^	2008	73	M	Asian	Anemia	None	0	Duodenum	ND	None	1 month (dead)
3	Gocho^9)^	2010	52	M	Asian	Perforation	None	0	Jejunum	ND	Surgery	12 months (dead)
4	Martínez Caselles^10)^	2010	57	M	Europian	Anemia	None	0	Duodenum	ND	ND	ND
5	Liu^11)^	2010	57	M	Asian	Intussusception	P/D, Adjuvant Chemotherapy (CDDP+PEM)	10	Jejunum	Ascending and descending colon, Mesenteric LN	Surgery	1 months (dead)
6	Navarro García^12)^	2015	67	M	Asian	Perforation	EPP, Adjuvant Chemotherapy	13	Jejunum	Mesenteric LN	Surgery	ND
7	Alkhayal^13)^	2016	65	M	Asian	Perforation	None	0	Jejunum	ND	Surgery	ND
8	Hamaoka^14)^	2021	72	M	Asian	Intussusception	CDDP+PEM, Nivo	18	Jejunum	Lung	Surgery, Chemotherapy	4 months (alive)
9	Our case	2023	87	M	Asian	Anemia, Abdominal pain	CDDP+PEM, CBDCA+PEM, GEM, Nivo	60	Jejunum	Mesenteric LN	Surgery	20 months (dead)

CBDCA, carboplatin; CDDP, cisplatin; EPP, extra-pleural pneumonectomy; GEM, gemcitabine; LN, lymph node; MPM, malignant pleural mesothelioma; ND, not described; Nivo, nivolumab; P/D, pleurectomy/decortication; PEM, pemetrexed

Diagnosis prior to symptom onset is challenging; most cases present with overt or occult bleeding, obstruction, or perforation. As suggested by Kakugawa et al., Gocho et al., and Navarro García et al., capsule endoscopy and DBE, complemented by PET/CT when feasible, facilitate earlier detection in MPM patients with unexplained anemia or abdominal pain.^7,9,12)^ In our case, CT prompted small-bowel evaluation and DBE confirmed an obstructive lesion; however, endoscopic biopsies were non-diagnostic, underscoring the role of surgical resection in selected cases for both diagnosis and symptom control.

Proposed routes of small-bowel metastasis include hematogenous and lymphatic spread as well as peritoneal dissemination. In our case, the absence of peritoneal carcinomatosis favored hematogenous or lymphatic routes. The presence of mesenteric lymph-node metastases adjacent to the jejunal lesion suggests lymphatic involvement; however, localized nodal enlargement does not exclude a hematogenous route.

The oncologic value of mesenteric lymph-node dissection remains uncertain. Among the nine published cases, lymph-node metastases were documented in three, including ours. Given the overall poor prognosis of MPM, the primary aims of surgery are symptom control (bleeding/obstruction) and definitive diagnosis. Lymph-node dissection may be reserved for patients with radiologically enlarged nodes or when preoperative distinction from primary small-bowel cancer is difficult—as in our case—and when the additional operative risk is acceptable.

At the time of resection, the tumor immunophenotype showed broad-spectrum cytokeratin positivity with CEA/BerEP4 negativity; calretinin and WT-1 were equivocal (focal/weak), which limits pathologic certainty. Where available, ancillary markers such as BAP1/MTAP and p16 FISH can support the diagnosis. In our case, BAP1/MTAP immunostaining and p16 FISH were not performed because of limited institutional availability; thus, the diagnosis relied on clinicopathological correlation, and a degree of diagnostic uncertainty remains. Notably, the immunophenotype of the jejunal lesion differed from that of the primary tumor, which may reflect spatial/clonal heterogeneity across tumor sites.

Although prolonged immune-checkpoint inhibition has been hypothesized to influence tumor morphology or antigen expression, the evidence is limited and causality cannot be established. An alternative—and potentially more likely—interpretation is that durable disease control and prolonged survival with nivolumab may have unmasked an uncommon metastatic site that might not have become clinically apparent in earlier eras with shorter survival. From a practical standpoint, this case highlights that rare metastatic presentations may be encountered more frequently among long-term survivors treated with ICIs, and new gastrointestinal symptoms such as abdominal pain, melena, or anemia should prompt timely evaluation even when the primary pleural lesion appears stable. In the ICI era, surgeons should be aware that such atypical metastatic presentations may require prompt surgical consultation for definitive diagnosis and symptom control.

## CONCLUSIONS

In MPM patients receiving ICI therapy, unexplained abdominal pain or gastrointestinal bleeding should prompt comprehensive gastrointestinal evaluation, including small-bowel assessment. When a resectable lesion is identified, surgical resection provides reliable symptom control and a definitive diagnosis, thereby supporting subsequent treatment planning.

## DECLARATIONS

### Availability of data and materials

The datasets supporting the conclusions of this study are included in this article and its additional files.
